# Functional Analysis of Conserved Motifs in the Mechanosensitive Channel Homolog MscS-Like2 from *Arabidopsis thaliana*

**DOI:** 10.1371/journal.pone.0040336

**Published:** 2012-06-29

**Authors:** Gregory S. Jensen, Elizabeth S. Haswell

**Affiliations:** Department of Biology, Washington University in Saint Louis, Saint Louis, Missouri, United States of America; USDA-ARS, United States of America

## Abstract

The Mechanosensitive channel of Small conductance (MscS) of *Escherichia coli* has become an excellent model system for the structural, biophysical, and functional study of mechanosensitive ion channels. MscS, a complex channel with multiple states, contributes to protection against lysis upon osmotic downshock. MscS homologs are widely and abundantly dispersed among the bacterial and plant lineages, but are not found in animals. Investigation into the eukaryotic branch of the MscS family is in the beginning stages, and it remains unclear how much MscS homologs from eukaryotes resemble *E. coli* MscS with respect to structure, function, and regulation. Here we test the effect of mutating three conserved motifs on the function of MscS-Like (MSL)2, a MscS homolog localized to the plastids of *Arabidopsis thaliana*. We show that 1) a motif at the top of the cytoplasmic domain, referred to here as the PN(X)_9_N motif, is essential for MSL2 function and for its proper intraplastidic localization; 2) substituting polar residues for two large hydrophobic residues located in the predicted pore-lining transmembrane helix of MSL2 produces a likely gain-of-function allele, as previously shown for MscS; and 3) mis-expression of this allele causes severe defects in leaf growth, loss of chloroplast integrity, and abnormal starch accumulation. Thus, two of the three conserved motifs we analyzed are critical for MSL2 function, consistent with the conservation of structure and function between MscS family members in bacteria and plants. These results underscore the importance of plastidic mechanosensitive channels in the maintenance of normal plastid and leaf morphology.

## Introduction

Mechanosensitive (MS) ion channels are membrane-embedded protein pores that open in response to mechanical force [1], [2]. Membrane tension can be transmitted directly to the channel from the lipid bilayer or conveyed indirectly through other cellular components [3]. MS channels have been identified in organisms from bacteria to humans, and are thought to provide a molecular mechanism for the cellular response to mechanical stimuli such as gravity, touch, sound, and osmotic pressure. The study of MS channels has medical relevance for the regulation of blood pressure, cardiac and muscular dysfunction, and the perception of sound and touch [1], [4].

**Figure 1:**
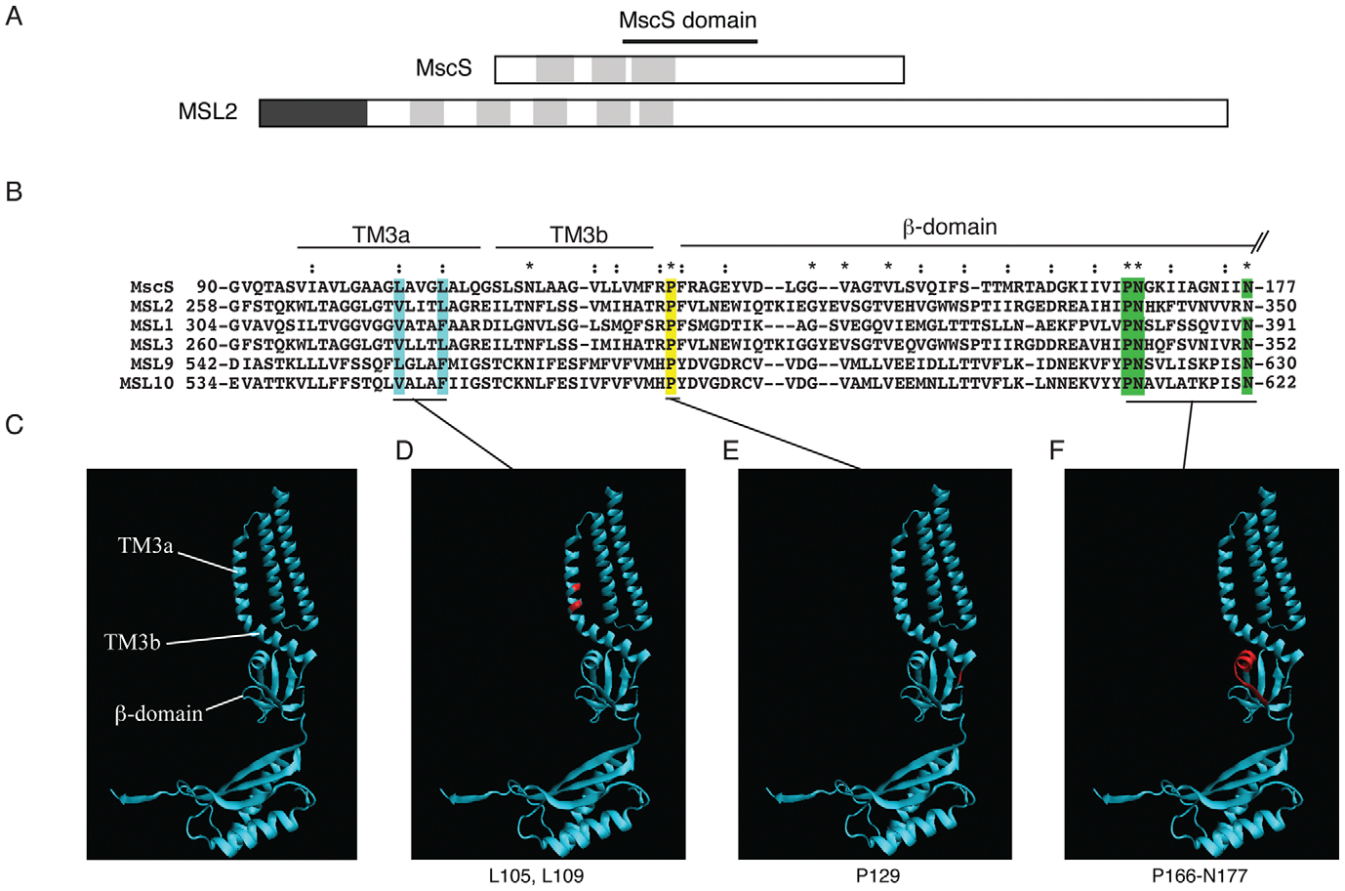
Identification of conserved motifs selected for functional analysis of MSL2. (A) Topology of MscS and predicted topology of MSL2. Dark grey box, chloroplast transit peptide. Light grey boxes, transmembrane helices. (B) The MscS domain according to an alignment of the conserved region of *E. coli* MscS and five representative MscS-Like (MSL) proteins from *A. thaliana*. Double dots and asterisks indicate similar and conserved residues, respectively. Sequences corresponding to TM3a, TM3b, and the ß-domain of MscS are indicated by lines [21]. (C–F) Ribbon diagram of a single subunit of MscS from the revised Rees crystal structure (PDB 20AU [13], [21]). The three conserved motifs functionally characterized in this report are highlighted in blue, (MscS L109, MSL2 V273/L277); yellow (MscS P129, MSL2 P297); and green (MscS P166/N167/N177; MSL2 P339/N340/N350) in (B) and shown in red in (D–F).

One of the best-studied MS channels is MscS (Mechanosensitive channel of Small conductance), an abundant, essentially nonselective channel found in the plasma membrane of *Escherichia coli* [5], [6], [7], [8], [9], [10]. MscS is gated directly through tension in the membrane [11], [12] and when open has a conductance of ∼1 nS with a proposed pore diameter of 10–15 Å [13], [14], [15], [16]. MscS, along with the Mechanosensitive channels of Large (MscL, ∼3 nS) and Mini conductance (MscM, ∼0.4 nS), promotes the survival of cells subjected to sudden osmotic downshock by allowing cytoplasmic osmolytes to exit as the cell swells [17], [18], [19], [20]. Two MscS crystal structures [13], [14], [21] along with biochemical and cell-based studies [22], [23] have established that MscS is a compact homoheptameric channel with a short extracellular domain, a transmembrane (TM) domain, and a relatively large (17 kDa) cytoplasmic domain comprised primarily of ß-sheets. Each MscS monomer contributes three helices to the transmembrane domain. The third of these, TM3, is split into two segments by a distinctive kink at G113, with TM3a forming the channel pore as it crosses the membrane, and TM3b lying almost parallel to the bilayer surface (see Figure 1C). Within the glycine- and alanine- rich environment of TM3a lie two larger hydrophobic residues, L105 and L109, which are proposed to form the hydrophobic seal or vapor lock that keeps the channel impermeable in the absence of membrane tension [14], [21], [24], [25]. The cytoplasmic cage comprises the middle ß-domain, the alpha/ß-domain, and a C-terminal ß-barrel. There is growing evidence that the cytoplasmic domain of MscS undergoes a conformational change when the channel is gated, perhaps indicating that it serves to regulate channel gating [26], [27], [28], [29], [30], [31], [32].

Homologs of MscS have been identified throughout bacterial and archaeal kingdoms. Many provide MS channel activity and/or function in osmotic stress protection [18], [33], [34], [35], [36], [37], though others do not [38], [39], [40], [41]. MscS homologs have also been identified in the genomes of a subset of eukaryotic lineages, including several fungal and all plant genomes examined to date [42], [43], [44], [45].

The region of MscS that is conserved among family members includes TM3 and the upper portion of the cytoplasmic domain [21], [42], [45], [46]. Outside of this region, MscS homologs vary considerably in size and topology: the number of TM helices ranges from 3 (as in MscS) to 11 (as in MscK) [22], [42], and large differences exist in the size of soluble domains (e.g., [18]). Though previous genetic studies have revealed a number of residues within the conserved region that are required for the stability or activity of MscS (summarized in [46], [27], [32], [47]), MscK [48], [49] and MscM [18], not all of the residues identified as conserved have been tested for functional relevance in MscS, nor has the functional relevance of any conserved residues from eukaryotic MscS proteins been reported.

Here we address the functional relevance of these conserved sequences in the *Arabidopsis thaliana* MscS-Like MS channel MSL2. MSL2 and close homolog MSL3 are localized to the plastid envelope, and they are required for normal plastid size and shape. Mutant plants lacking functional MSL2 and MSL3 have variegated leaves, enlarged chloroplasts, and large, spherical leaf epidermal plastids that are unable to adjust their volume in response to extreme osmotic shock [50], [51], [52]. MSL2 has five predicted TM helices and an extensive C-terminal domain predicted to be in the stroma (Figure 1A). In this report, we identify the three motifs within the conserved region that are most conserved between *E. coli* MscS and *Arabidopsis* MSLs and evaluate their contribution to the *in planta* function of MSL2.

## Results

### Identification of conserved motifs in *Escherichia coli* MscS and five *Arabidopsis thaliana* MscS-Like proteins

Using ClustalX alignment software [53], we identified the region of highest conservation between MscS and its *Arabidopsis* homologs, referred to here as the MscS domain (Figure 1A). Figure 1B shows a protein sequence alignment of the ∼90 amino acid MscS domain from MscS and five MscS-Like (MSL) proteins. This region was previously identified as conserved among MscS homologs [42], [45] and contains the “TM3 module of MscS for Bacteria and Eukarya” [46]. Mapped onto the crystal structure of MscS, this sequence comprises the channel-forming helix TM3, and 50 amino acids of the upper portion of the cytoplasmic domain, termed the middle ß-domain ([21], Figure 1B). In MSL2, this sequence corresponds to the fifth predicted transmembrane helix and 54 adjacent amino acids that are predicted to reside in the plastid stroma [50]. Within this conserved MscS domain, we selected three smaller regions of conserved residues for functional analysis (Figure 1B–F).

The first motif selected for analysis was a pair of large hydrophobic residues in the channel-forming helix (Figure 1D). In MscS, these residues (L105 and L109) are predicted to provide the tightest constriction when the channel is closed [14], [21]. Though there is no strict conservation of amino acid identity in this region, some MSLs exhibit the general pattern (small hydrophobic residues interspersed with large hydrophobic residues) that has been described previously for MscS [25]. The L109S mutant is a gain-of-function (GOF) lesion, lethal when expressed in *E. coli* [22] and showing increased open probability at low pressures by patch-clamp electrophysiology [25]. We therefore chose to characterize the effect of substituting serine for V273 and/or L277, the two residues that best align with L105 and L109, in MSL2.

The second motif selected for analysis was a conserved proline that resides at the junction between the pore-lining helix and the soluble cytoplasmic domain (Figure 1E). This residue (P129 in MscS and P297 in MSL2) was identified as conserved among MscS family members [46], but has not been functionally studied to date in MscS or any other homolog. In MscS, two residues in this region, R128 and R131, have been proposed to serve as lipid interacting anchors that resist rotation of TM3b during opening of the channel [54] or provide electrostatic interactions between the cytoplasmic domain and the TM1-TM2 connecting loop [29], [55].

The third motif selected for analysis was a set of three highly conserved residues, a proline and two asparagines, located in a short alpha helix at the top of the ß-domain and in close proximity to the pore-lining helix in the crystal structure (Figure 1F). This PN(X)_9_N motif (P166/N167/N177 in MscS and P339/N340/N350 in MSL2) was identified as well-conserved in most families by Pivetti and colleagues with percent identities of 96%, 86%, and 68% for the proline and two asparagines, respectively [42]. N167I and N167Y were previously identified as loss-of-function (LOF) lesions in a screen for potassium-leaking mutants of MscS [32].

### Leaf morphology phenotypes in *msl2-3* plants expressing MSL2 V273S/L277S, P297A, and P339A/N340A/N350A

**Figure 2:**
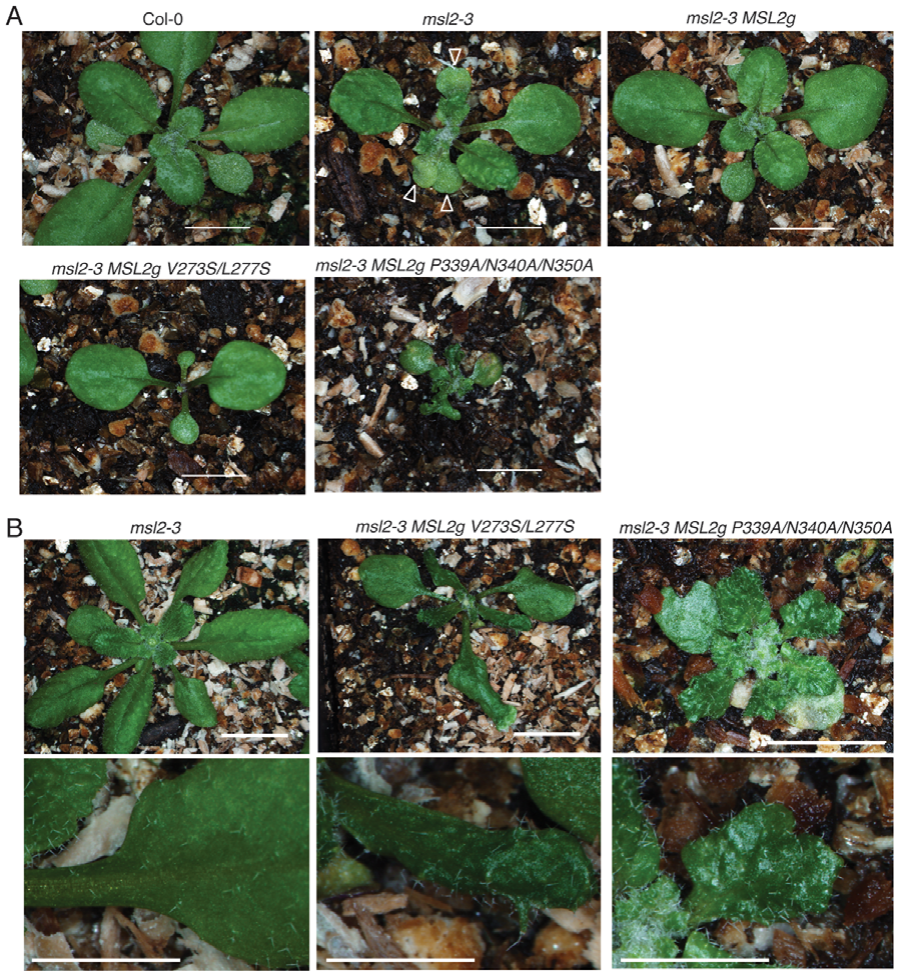
*msl2-3* plants expressing *MSL2g V273S/L277S* or *MSL2g P339A/N340A/N350A* exhibit dramatic defects in leaf morphology. (A) Representative images of 2 week-old *msl2-3* T2 plants transformed with *MSL2g* variants. Bar is 5 mm. Arrows indicate triple cotyledons in the *msl2-3* mutant. (B) Representative images of 3 week-old plants from the indicated lines. Top, bar is 8 mm; bottom, size bar is 4 mm.

We chose *Arabidopsis* MSL2 as a starting point for the functional characterization of these conserved motifs because we recently identified a null allele of *MSL2*, *msl2-3*, that has an easily identified leaf morphology phenotype [52]. *msl2-3* mutant plants are slightly smaller than wild type, and young leaves exhibit notched edges and a rumpled surface. These phenotypes are rescued by expression of the *MSL2g* transgene, which contains the wild type *MSL2* gene in its genomic context and confers resistance to the herbicide glufosinate [50], [52]. Site-directed mutagenesis was used to produce mutant versions of the *MSL2g* transgene, and they were introduced into either wild type Columbia (Col-0) or *msl2-3* plants by *Agrobacterium*-mediated transformation [56]. Ten independent T2 (second transgenic generation) lines were characterized for each transgene, and lines exhibiting approximately 75% glufosinate resistance, consistent with a single *MSL2g* insertion event, were identified for further analysis. The results of our initial analysis of leaf morphology are summarized in Table 1 and representative plant images presented in Figure 2.

**Table 1:** Phenotypic characterization of T2 lines expressing MSL2g variants in the *msl2-3* and the wild type Columbia (Col-0) backgrounds.

			# of T2 lines with phenotype			
Back-ground	Transgene	Total number of T2 lines with single transgene	Wild type	Narrow, developmentally delayed leaves	Variegated, notched leaves	Slightly irregular leaf margins
msl2-3	MSL2g	6	6	0	0	0
	MSL2g V273S/L277S	8	1	7 (95)	0	0
	MSL2g P297A	9	9	0	0	0
	MSL2g P339/N340A/N350A	10	0	0	10 (93)	0
Col-0	MSL2g	7	7	0	0	0
	MSL2g V273S/L277S	8	2	6 (28)	0	0
	MSL2g P339/N340A/N350A	9	6	0	0	3 (37)

Ten independent T2 lines were analyzed from each transgene. Data is presented as number of T2 lines exhibiting each phenotype. The percentage of glufosinate-resistant seedlings exhibiting each phenotype is presented in parentheses.

As expected, *msl2-3* plants showed rumpled, notched leaves, and frequently exhibited multiple cotyledons (Figure 2A, arrows) while *msl2-3* plants stably transformed with the wild type *MSL2g* transgene resembled wild type plants. All *msl2-3 MSL2g P297A* lines were indistinguishable from wild type, indicating that mutating this residue has no (or a very subtle) effect on MSL2 function (Table 1). However, neither the *MSL2g V273S/L277S* nor the *MSL2g P339A/N340A/N350A* transgenes produced wild type phenotypes in the *msl2-3* mutant background. We observed a novel phenotype in *msl2-3 MSL2g V273S/L277S* T2 lines; though the cotyledons and first two leaves appeared normal, later leaves were extremely slow growing, and developing leaf primordia were small and narrow (Table 1, Figure 2A). Later in development, *msl2-3 MSL2g V273S/L277S* plants had leaves that were dark green, narrow, and misshapen, but the plants were otherwise healthy and fertile (Figure 2B). This phenotype was also observed in the Col-0 background (Table 1), indicating that it is dominant. These results are consistent with the production of a GOF phenotype generated by the introduction of polar residues into the pore-lining helix of the channel, as previously observed with MscS L109S [22], [25]. All ten *msl2-3 MSL2g P339A/N340A/N350A* T2 lines examined exhibited severe leaf notching and variegation and small stature both early and late in development (Figure 2A and B). In the Col-0 background, *MSL2g P339A/N340A/N350A* transgene only rarely produced a subtle phenotype, slightly irregular leaf margins (Table 1), indicating that this allele is largely recessive.

### Plastid morphology phenotypes in *msl2-3* plants expressing MSL2 V273S/L277S, MSL2 and MSL2 P339A/N340A/N350A

**Figure 3:**
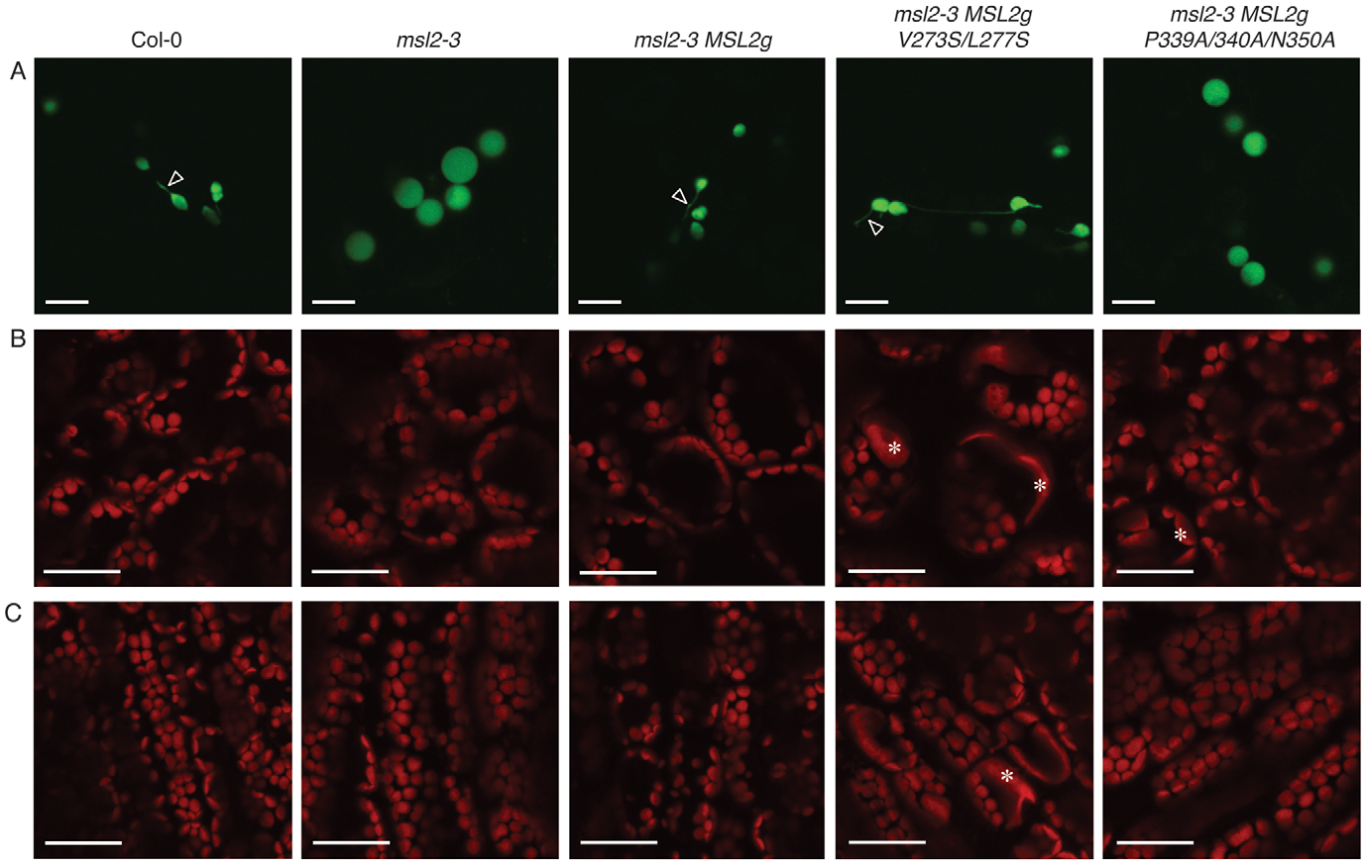
Expressing MSL2g mutants in the *msl2-3* background affects plastid morphology. (A) Confocal laser scanning microscopy images of leaf epidermal cells transiently expressing the fluorescent stromal marker RecA-YFP. Arrows indicate stromules. Results are representative of at least 50 cells per genotype. Size bar is 10 microns. (B, C) Representative CLSM images of mesophyll cells from the leaf blade (B) and petiole (C) of 3 week-old T2 plants. Chlorophyll autofluorescence is pseudocolored red. In (B), the same region of each leaf (the lower right quadrant, midway between the vasculature and the leaf margin) was imaged. Asterisks mark enlarged chloroplasts. Size bar in (B) and (C) is 30 microns.

As MSL2 had been previously implicated in the control of plastid size and shape [50], we investigated the morphology of chloroplasts and leaf epidermal plastids in *MSL2g* variant T2 lines. To visualize leaf epidermal plastids, we used particle bombardment to induce transient expression of the plastid-targeted fluorophore RecA-YFP, allowing us to image leaf epidermal plastids present in the epidermis of rosette leaves from wild type, mutant, and mutant transgenic lines. Confocal laser scanning microscopy (CLSM) of bombarded cauline leaves showed that, while 100% of the wild type plastids (n = 50 cells) were ovoid in shape and often had visible stromules (Figure 3A, arrows), 77% of the cells examined from the *msl2-3* mutant harbored only large, spherical leaf epidermal plastids that did not exhibit stromules (n = 64). This phenotype was similar to that previously described in *msl2-1 msl3-1* mutants [50], [51]. The presence of the wild type *MSL2g* transgene rescued this phenotype, producing leaf epidermal plastids with wild type morphology in 92% of the cells examined (n = 53); while the presence of *MSL2g P339A/N340A/N350A* did not, producing only round plastids in 79% of the cells examined (n = 68). The *MSL2g V273S/L277S* transgene partially rescued the round plastid phenotype, as T2 lines harboring this transgene exhibited ovoid plastids in 64% of the cells examined, and a further 27% of the cells examined contained a mixture of ovoid and round plastids (n = 55).

CLSM was also used to characterize the morphology of chloroplasts in mesophyll cells of the blade (Figure 3B) and petiole (Figure 3C) of young rosette leaves of wild type, *msl2-3*, and T2 lines harboring *MSL2g* variants in the *msl2-3* background. As shown, *msl2-3* plants have normally sized and shaped chloroplasts and chloroplast morphology was not appreciably changed by expression of wild type *MSL2g* in this background. However, slightly enlarged chloroplasts were occasionally found in *msl2-3 MSL2g P339A/N340A/N350A* plants, and severely enlarged chloroplasts were abundant in both types of mesophyll cells in *msl2-3 MSL2g V273S/L277S* T2 plants (Figure 3B and C, asterisks). These results implicate residues in both the pore-lining helix and the cytoplasmic domain in the regulation of chloroplast size by MSL2.

### Mis-expression of MSL2-YFP variants

**Figure 4:**
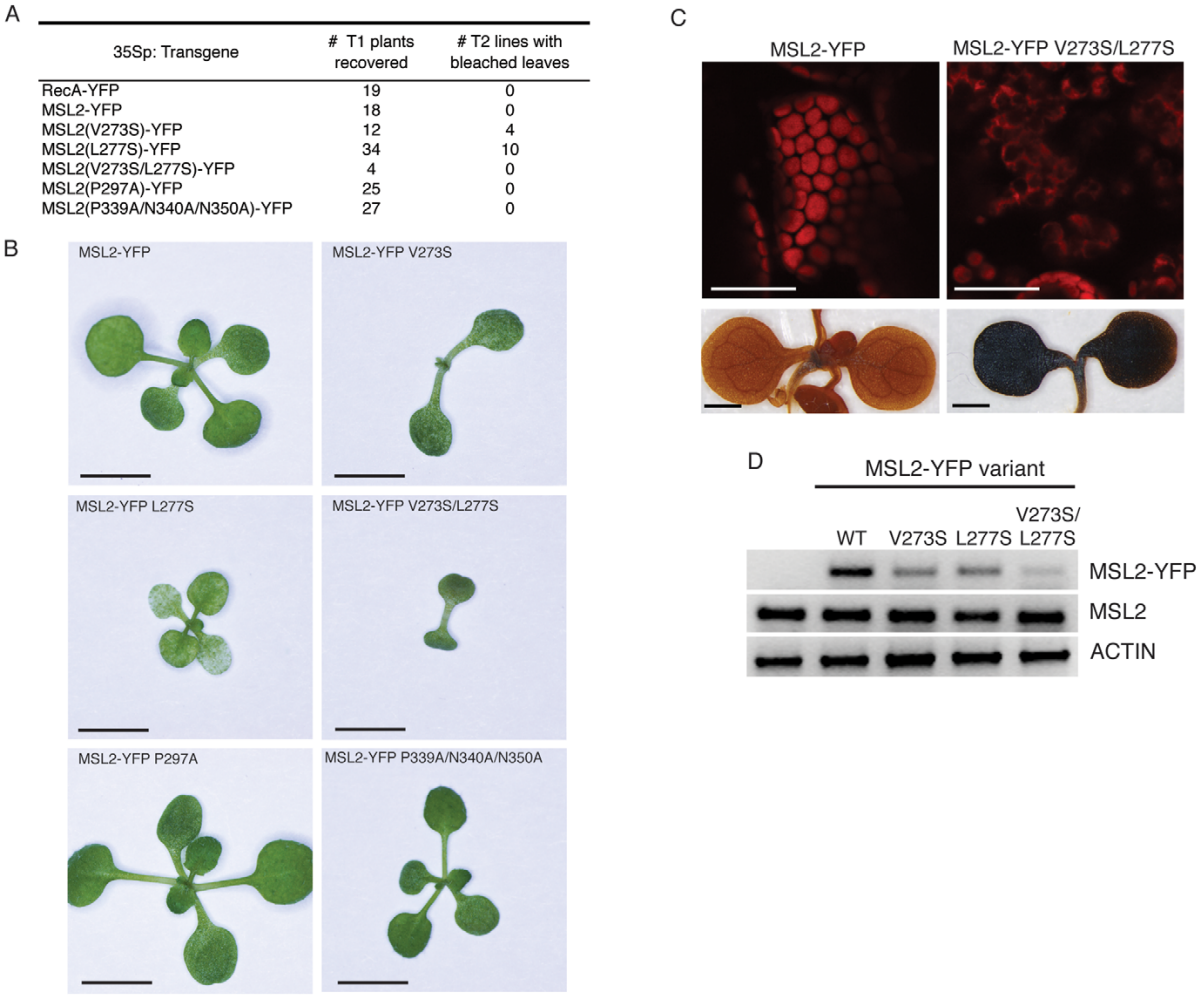
Mis-expression of MSL2-YFP V273S, L277S or V273S/L277S causes severe defects in leaf growth and chloroplast integrity. (A) Number of T1 plants recovered from side-by-side plant transformations and phenotypes of subsequent T2 lines. (B) Representative images of 2 week-old T2 seedlings. Size bar is 4 mm. (C) Top, representative confocal scanning images of cotyledons of the indicated T2 seedlings. Chlorophyll autofluorescence is pseudocolored red. Size bar is 30 microns. Bottom, representative T2 seedlings stained with Lugol's reagent to indicate starch content. Scale bar is 1 mm. Note the loss of all leaf primordial, as frequently observed in these lines. (D) Agarose gel of PCR products specific to the transgenes and genes indicated to the right. cDNA derived from the indicated T2 lines shown in (D) was used as template.

To further investigate the function of MSL2 V273S/L277S, P297A, and P339A/N340A/N350A mutants, we used site-directed mutagenesis to introduce these lesions into a previously described construct wherein expression of an MSL2-YFP fusion protein is driven by the constitutive Cauliflower mosaic virus 35S promoter [50], [57]. An *MSL2-GFP* fusion has been shown to complement the mutant *msl2 msl3* variegated leaf phenotype when expressed under the control of the *MSL2* promoter, indicating that the fusion protein is functional [50]. Mis-expression of RecA-YFP, MSL2-YFP, MSL2-YFP P297A, or MSL2-YFP P339A/N340A/N350A did not affect plant morphology (Figure 4A and B). However, strong phenotypes were observed in plants mis-expressing MSL2-YFP variants harboring polar substitutions in the pore-lining helix. Approximately one third of the T1 lines mis-expressing MSL2-YFP V273S or MSL2-YFP L277S exhibited small seedlings with patchy white and green cotyledons; these seedlings frequently arrested with small, deformed leaf primordia. Only four T1 seedlings mis-expressing MSL2-YFP V273S/L277S were recovered; their progeny were severely stunted and no true leaves or leaf primordia were detectable. To characterize gene expression in these lines, cDNA was prepared from whole seedling mRNA isolated from the T2 plants imaged in Figure 4B. Products specific to the *MSL2-YFP* variant transgenes, the endogenous *MSL2* gene, and an *ACTIN* control were PCR amplified from these cDNAs. As Figure 4D shows, low levels of *MSL2-YFP V273S, L277S*, and *V273S/L277S* transgene expression were detected in the transgenic lines, while no change in the endogenous *MSL2* expression was observed. Because the levels of transgene expression appear lower than the endogenous *MSL2* gene when measured in the whole leaf, we refer to this as mis-expression rather than over-expression. These data indicate that the observed phenotypes cannot be attributed to silencing of the endogenous *MSL2* gene, and that the *MSL2-YFP V273S, L277S*, and *V273S/L277S* alleles are expressed at low levels in the recovered lines. Inviability of lines expressing higher levels of V273S/L277S may explain the low number of T1 lines recovered.

The bleached or patchy leaves observed in T1 and T2 lines mis-expressing MSL2-YFP V273S/L277S suggested that there might be a defect in chloroplast function. We therefore inspected chloroplasts present in the cotyledons of arrested T2 seedlings mis-expressing MSL2-YFP V273S/L277S using CLSM. Unlike chloroplasts from plants expressing MSL2-YFP, which resembled the wild type with smooth, rounded edges and strong chlorophyll fluorescence, chloroplasts from plants mis-expressing MSL2-YFP V273S/L277S were severely deformed (Figure 4 C, top panel). In these lines, the chloroplasts appeared to lose integrity and exhibited uneven edges and large black voids. We hypothesized that the black voids–regions of the stroma inaccessible to chlorophyll–might indicate the presence of large starch granules. Indeed, starch staining of seedlings with Lugol's reagent revealed a striking increase in starch content in the MSL2-YFP V273S/L277S T2 seedlings (Figure 4C, bottom panel) compared to mis-expression of wild type MSL2-YFP.

### MSL2-YFP P339A/N340A/N350A shows aberrant intraplastidic localization

**Figure 5:**
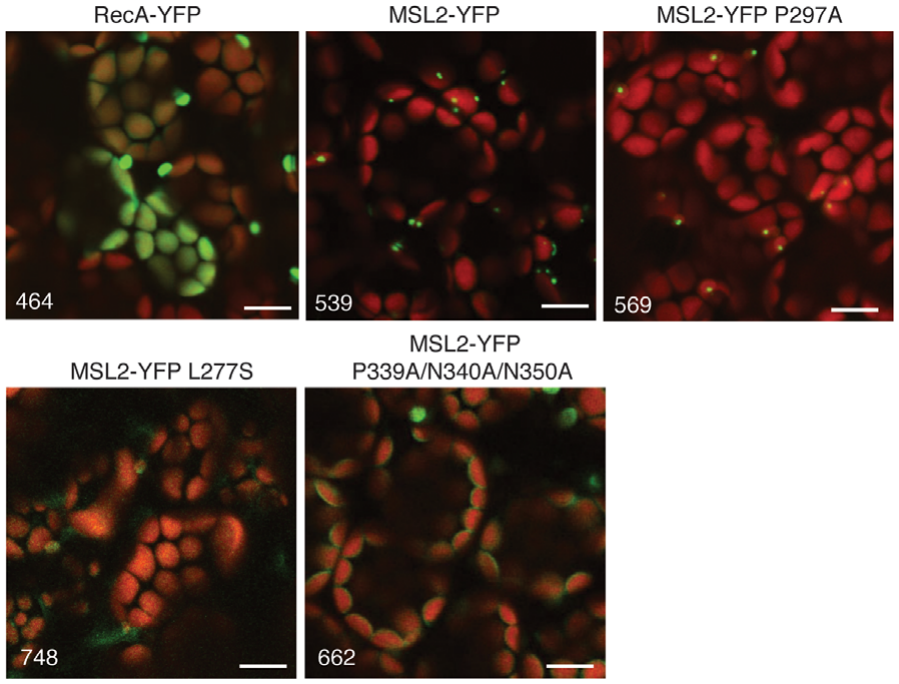
MSL2-YFP P339A/N340A/N350A shows aberrant intraplastidic localization. Representative confocal images of rosette leaves from T3 plants expressing the indicated transgenes, with the YFP channel voltage for each image shown in the lower left hand corner. The same region of the leaf was imaged as in Figure 3B. YFP signal is pseudo-colored green and chlorophyll is pseudo-colored red. Size bar is 10 microns.

MSL2 and MSL3 show an intriguing localization pattern to puncta within the chloroplast envelope [50]. Localization to the puncta is observed at high and low levels of expression but its functional relevance remains unknown. We used CLSM to characterize the intraplastidic localization of MSL2-YFP variants mis-expressed in the wild type background in the T2 generation. While the expression level of MSL2-YFP L277S was too low to distinguish from background (as predicted from RT-PCR, Figure 4D), and the localization of MSL2-YFP P249A was indistinguishable from the wild type, we consistently observed a loss of localization to puncta in the MSL2-YFP P339A/N340A/N350A T2 lines (Figure 5). In leaf mesophyll cells mis-expressing MSL2-YFP P339A/N340A/N350A, YFP signal was localized uniformly to the periphery of the chloroplast, indicating that the mutant protein is stable and targeted to the chloroplast envelope, but that it lacks the ability to form puncta.

## Discussion

While MscS is an excellent model for the study of MS channels, its large family of homologs provides a new opportunity to study MS channels in the context of a multi-cellular eukaryote. Though several reports have identified the domain conserved among MscS family members [42], [43], [45], [46], the functional relevance of these sequences has not been directly addressed in any eukaryotic family members. We have therefore taken advantage of the experimental strengths of the plastid-localized MscS homolog from *Arabidopsis thaliana* MSL2 to characterize the *in planta* effect of mutating the three most highly conserved motifs: 1) the presumptive hydrophobic seal residues V273 and L277, that lie within the pore-lining TM helix; 2) the conserved P297 at the end of the TM domain; and 3) the PN(X)_9_N motif in the ß-domain at the top of the soluble C-terminal domain (Figure 1). MSL2 variants carrying lesions in these motifs were either expressed at endogenous levels in both *msl2-3* null and wild type Columbia (Col-0) backgrounds, or mis-expressed as YFP fusion proteins in the Col-0 background. We then characterized the effect of these lesions on plant and plastid morphology, as well as on intraplastidic localization of MSL2. While changing the conserved P297 to alanine had no detectable effect on MSL2 function in any of these assays, we found that motifs in the pore-lining helix and in the cytoplasmic domain are essential for normal MSL2 function.

### The PN(X)_9_N motif is important for MSL2 function *in planta*

The PN(X)_9_N motif located at the top of the soluble cytoplasmic domain is critical for MSL2 function. Though YFP fusion experiments indicate that MSL2 P339A/N340A/N350A is stable (Figure 5), it lacks function *in planta* (Table 1, Figures 2 and 3). Expressing *MSL2 P339A/N340A/N350A* from its own promoter did not rescue the *msl2-3* mutant leaf or plastid morphology, instead producing more severe defects in plant stature and leaf notching than observed in the null mutant. We thus suspect that MSL2 P339A/N340A/N350A is capable of interfering with the function of other MscS homologs, most likely MSL3, in the absence of wild type MSL2. Electrophysiological data suggests that MSL9 and MSL10, two Arabidopsis MscS homologs expressed in the root plasma membrane, form heteromeric channels [58], [59]. Consistent with this hypothesis, the presence of *MSL2g P339A/N340A/N350A* in the Col-0 background (when the wild type *MSL2* gene would be present and capable of contributing normally functioning subunits to both homomeric and heteromeric channels) had only occasionally a mild phenotypic effect (Table 1, Figure 4).

In the MscS crystal structure the PN(X)_9_N motif is located at the top of the cytoplasmic cage, a strategic position for interaction with the TM3b helix (Figure 1F, [21]). Among several recently identified and characterized MscS mutants that support such an interaction are two lesions in N167 (N167Y and N167I) that exhibit a LOF phenotype and produce a fast inactivating channel activity [32]. These data were interpreted to suggest direct binding between TM3b and the ß-domain, that when disrupted results in channel inactivation. If MSL2 has a similar pore and ß-domain structure as MscS, then MSL2 P339A/N340A/N350A could be non-functional due to the disruption of strong binding between TM3b and the ß-domain and/or the enhancement of channel inactivation.

### The PN(X)_9_N motif of the ß-domain is required for proper intraplastidic localization of MSL2-YFP

Analysis of the intraplastidic localization of MSL2-YFP P339A/N340A/N350A indicated that this variant was produced at levels similar to that of wild type MSL2-YFP and that it was properly targeted to the chloroplast envelope (Figure 5). Unexpectedly, MSL2 P339A/N340A/N350A did not localize to puncta at the poles of chloroplasts like the wild-type protein, but was evenly distributed throughout the chloroplast envelope. Neither the mechanism by which MSL2 localizes to puncta on the chloroplast envelope nor the functional relevance of this localization pattern is known. However, these data establish a correlation between localization and function, and we speculate that localization of MSL2 to puncta is sensitive to channel function and/or conformation.

### Polar substitutions in the predicted pore-lining helix of MSL2 generate a likely gain-of-function (GOF) allele

Gain-of-function (GOF) mutations alter a gene product to produce a new or abnormal function, and are usually dominant. Introducing polar residues at or upstream of the hydrophobic seal of MscS or MscK leads to a GOF phenotype (cell death) and channels that are easier to gate [16], [22], [25], [32], [37], [47], [48], [49]. Serine substitutions in the two residues of MSL2 that best align with the hydrophobic seal residues of MscS also produced a dominant allele with novel functions. We note however that the precise identification of hydrophobic seal residues in MSL2 awaits structural information about its transmembrane pore-forming domain.

The data shown in Table 1, Figure 2 and Figure 3 provide evidence that MSL2 V273S/L277S has new or abnormal functions. Expressing MSL2 V273S/L277S from the endogenous promoter in either the *msl2-3* null or the wild type background produced plants with dominant leaf morphology defects that did not resemble (and were not simply more severe than) the *msl2-3* LOF. In addition, MSL2 V273S/L277S was partially able to rescue the large, round phenotype of the *msl2-3* mutant; while the leaf epidermal plastids in *msl2-3* plants are mostly large and round, 91% of the cells from in *msl2-3* plants harboring *MSL2g V273S/L277S* had some (or all)plastids with wild type morphology. These data are consistent with a model wherein MSL2 V273S/L277S is a functional channel capable of releasing plastidial osmotic stress [51] but is inappropriately active, creating defects in leaf and plant morphology.

Confirmation of this model awaits identification of a system for the electrophysiological analysis of MSL2. To date, we have been unable to identify a MS channel activity associated with MSL2 expressed in *Xenopus* ooctyes or in *E. coli* spheroplasts. In addition, the presence of *MSL2g V273S/L277S* was associated with a phenotype that is not obviously associated with a GOF allele. *msl2-3* plants expressing *MSL2g V273S/L277S* exhibited grossly enlarged chloroplasts, a phenotype similar to that observed in *msl2 msl3* double LOF mutants (Figure 3, [52]). We have shown that the large chloroplasts observed in *msl2 msl3* double mutants are likely the result of overactive FtsZ ring assembly, though the mechanism by which MS channels influence plastid division site selection is not known [52], [60]. It has previously been observed that over- and under-expression of established plastid division proteins can produce enlarged plastids (for a recent example, see [61]). We thus speculate that a version of *MSL2* that releases membrane tension too easily might have the same phenotypic effect as an *msl2* LOF mutant if precise plastid ion concentration or membrane tension is required for normal regulation of FtsZ assembly.

### Mis-expression of MSL2 variants with lesions in the pore-lining helix causes severe defects in seedling viability and chloroplast morphology and function

Mis-expressing MSL2-YFP V273S, L277S, or V273S/L277S in a wild type background caused developmental arrest of seedlings at the cotyledon stage (Figure 4). The rare plants that survived exhibited cotyledons with chlorotic patches, abnormally shaped chloroplasts and the over-accumulation of starch. As none of these phenotypes were seen in plants mis-expressing wild type MSL2-YFP, nor in *msl2-3* null mutants, these data provide further evidence for the importance of V273 and L277 in the normal function of MSL2, and are consistent with our hypothesis that MSL2 V273S/L277S is a GOF mutant. These phenotypes were not observed in MSL2g V273S, L277S plants, and considering the low levels of expression of the MSL2-YFP V273S/L277S transgene indicated in Figure 4D, they cannot be explained by protein-level defects such as defects in assembly, localization, or aggregation. We speculate that inappropriate activation of the MSL2 channel leads to altered concentrations of osmolytes in the stroma, which would broadly affect chloroplast integrity, sugar partitioning, and other essential plastid functions [62].

Taken together, the data presented here show that two of three conserved motifs we characterized are important for the function of MSL2, a plastid-localized MscS homolog from *Arabidopsis*. Until plastid-localized MscS family members can be successfully studied by patch clamp electrophysiology, a shared requirement for conserved residues constitutes the best evidence that MSL2 operates with the same molecular mechanism as MscS. Furthermore, these studies validate previously published phylogenetic analyses and illustrate the advantages of including eukaryotic MscS homologs proteins in the study of mechanosensitive channels.

## Materials and Methods

### Alignment and ribbon diagrams

Alignment of the conserved regions of *Escherichia coli* MscS, and *Arabidopsis thaliana* MSL1 (At4g00290), MSL2 (At5g10490), MSL3 (At1g58200), MSL9 (At5g19520) and MSL10 (At5g12080) proteins was performed using ClustalX 2.1. Ribbon diagrams were generated with Visual Molecular Dynamics freeware using the revised version of the MscS coordinates (PDB 20AU, [13]).

### Plasmids and bacterial strains

Point mutations were introduced into pBGW-MSL2g and MSL2-YFP [50] by site-directed mutagenesis. Oligos encoding the relevant base pair changes (listed in Table S1) were combined with 5–10 ng plasmid DNA (isolated from the *dam+* NEB Turbo *E. coli* strain) and subjected to 18 cycles of PCR amplification. PCR products were then digested with DpnI and transformed into competent NEB Turbo *E. coli*. Positive clones were identified by restriction enzyme digestion when possible and then sequenced. RecA-YFP was generated from MSL3-YFP by replacing the MSL3 open reading frame with the chloroplast targeting sequence from RecARED [50].

### Plant growth

The *msl2-3* allele, which was obtained from the GABI collection, is in the Columbia (Col-0) background [63]. *MSL2g* transgenes were selected in the *msl2-3* or the Col-0 background in both T1 and T2 generations by sowing on soil and spraying with 0.1% glufosinate (sold as Finale by Bayer). MSL2-YFP variants and RecA-YFP transgenes were selected in the Col-0 background in the T1, T2, and T3 generations on 1X MS Hygromycin plates (4.33 g/L Murashige and Skoog salts (Caisson Labs) and 8 g/L micropropagation agar (Caisson Labs) supplemented with 0.3% sucrose and 35 mg/L Hygromycin). All plants were grown in 16 hrs of light at 20–22°C and light fluence from 150–195 µmoles m^−1^ sec^−1^.

### Microscopy

#### Light microscopy of seedlings

1 or 3 week-old seedlings grown on soil or 10 day-old seedlings grown on plates were photographed with an Olympus SZX7 dissecting scope attached to the Olympus DP71 color digital camera and images captured with DP71 software.

#### Confocal laser scanning microscopy (CLSM) of chloroplasts and plastids

All CLSM was performed using the Fluoview FV-1000 (Olympus), and images were captured with FVIO-ASW software. Chlorophyll autofluorescence was excited at 635 nm and emissions collected with a 655- to 755-nm band-pass filter. Transient expression of RecA-YFP was induced by bombardment of *Arabidopsis* cauline leaves as described in [50], except that 3.25 mg of gold particles were coated with 12 µg of 0.5 mg/ml RecA-YFP in the presence of 108 µl 2.5 M CaCl_2_ and 48 µl 0.1 M spermidine, washed once with 70% ethanol, and resuspended in 100% ethanol before application to carrier discs. For imaging of MSL2-YFP–expressing lines and transient expression of RecA-YFP localized to leaf epidermal plastids, YFP signal was excited at 515 nm and emissions collected with a 535- to 565-nm band-pass filter.

### RT-PCR

Seedlings growing on MS Hygromycin plates were pooled and RNA isolated using Trizol reagent as directed by the manufacturer. *ACTIN*, *MSL2*, and the *MSL2-YFP* transgene sequences were amplified from cDNA using the oligos listed in Table S1. PCR products were separated on a 1% agarose gel and imaged with ethidium bromide.

### Starch Staining

Seedlings were placed in 80% ethanol at 80 degrees for 15 minutes to decolorize. All ethanol was removed and replaced with Lugol's solution (5.7 mM iodine, 43.4 mM KI). After incubation at room temperature for 15 min, seedlings were washed in distilled water and mounted on slides in water for imaging as described for seedlings above.

## Supporting Information

Table S1. Oligonucleotides used in this work.
(PDF)
