# Supplementary material for: Functional Analysis of Conserved Motifs in the Mechanosensitive Channel Homolog MscS-Like2 from Arabidopsis thaliana
Source: PLoS One. 2012 Jun 29;7(6):e40336. doi: 10.1371/journal.pone.0040336 (PMC3386975; doi:10.1371/journal.pone.0040336)
Supplement: Table S1. — Oligonucleotides used in this work. (PDF) [file pone.0040336.s001.pdf]

Table S1. Oligonucleotides used in this work

Oligo name	Gene	Use	Base Pair Sequence
P297A	*MSL2*	site directed mutagenesis	CATGCAACCCGGGCATTTGTTTTGAATGAGTGGATTC
P297A-GC	*MSL2*	site directed mutagenesis	GAATCCACTCATTCAAAACAAATGCCCGGGTTGCATG
V273S	*MSL2*	site directed mutagenesis	GCTGGAGGTCTTGGGACAAGTTTGATTACTCTTGCTGGCCG
V273S-GC	*MSL2*	site directed mutagenesis	CGGCCAGCAAGAGTAATCAAACTTGTCCCAAGACCTCCAGC
L277S	*MSL2*	site directed mutagenesis	GGTCTTGGGACAGTTTTGATTACTAGTGCTGGCCGAGAG
L277S-GC	*MSL2*	site directed mutagenesis	CTCTCGGCCAGCACTAGTAATCAAAACTGTCCCAAGACC
VL2S	*MSL2*	site directed mutagenesis	GGTCTTGGGACAAGTTTGATTACTAGTGCTGGCCGAGAG
VL2S-GC	*MSL2*	site directed mutagenesis	CTCTCGGCCAGCACTAGTAATCAAACTTGTCCCAAGACC
PNN2A	*MSL2*	site directed mutagenesis	CACATCGCAGCCCATAAGTTCACAGTTAATGTCGTGAGAGCTCTTACTC
PNN2A-GC	*MSL2*	site directed mutagenesis	GAGTAAGAGCTCTCACGACATTAACTGTGAACTTATGGGCTGCGATGTG
Actin.F2	*ACTIN*	RT-PCR	TACGCCAGTGGTCGTACAAC
Actin.R2	*ACTIN*	RT-PCR	TGTGAGACACACCATCACCAGA
10490.F3	*MSL2*	RT-PCR	GAGAACACTTCCAATCGAAGAAGAGATAC
10490.R2	*MSL2*	RT-PCR	CGGCTCGGTTGAAGCACC
GFP_UP	*GFP*	RT-PCR	CTCGCCGGACACGCTGAACTTGT
